# Supplementary figures and images for: Pan-cancer multi-omics characterization of calcyphosine and its revealed links to the immune microenvironment and regulatory networks in endometrial carcinoma
Source: Front Immunol. 2025 Nov 26;16:1688606. doi: 10.3389/fimmu.2025.1688606 (PMC12689553; doi:10.3389/fimmu.2025.1688606)

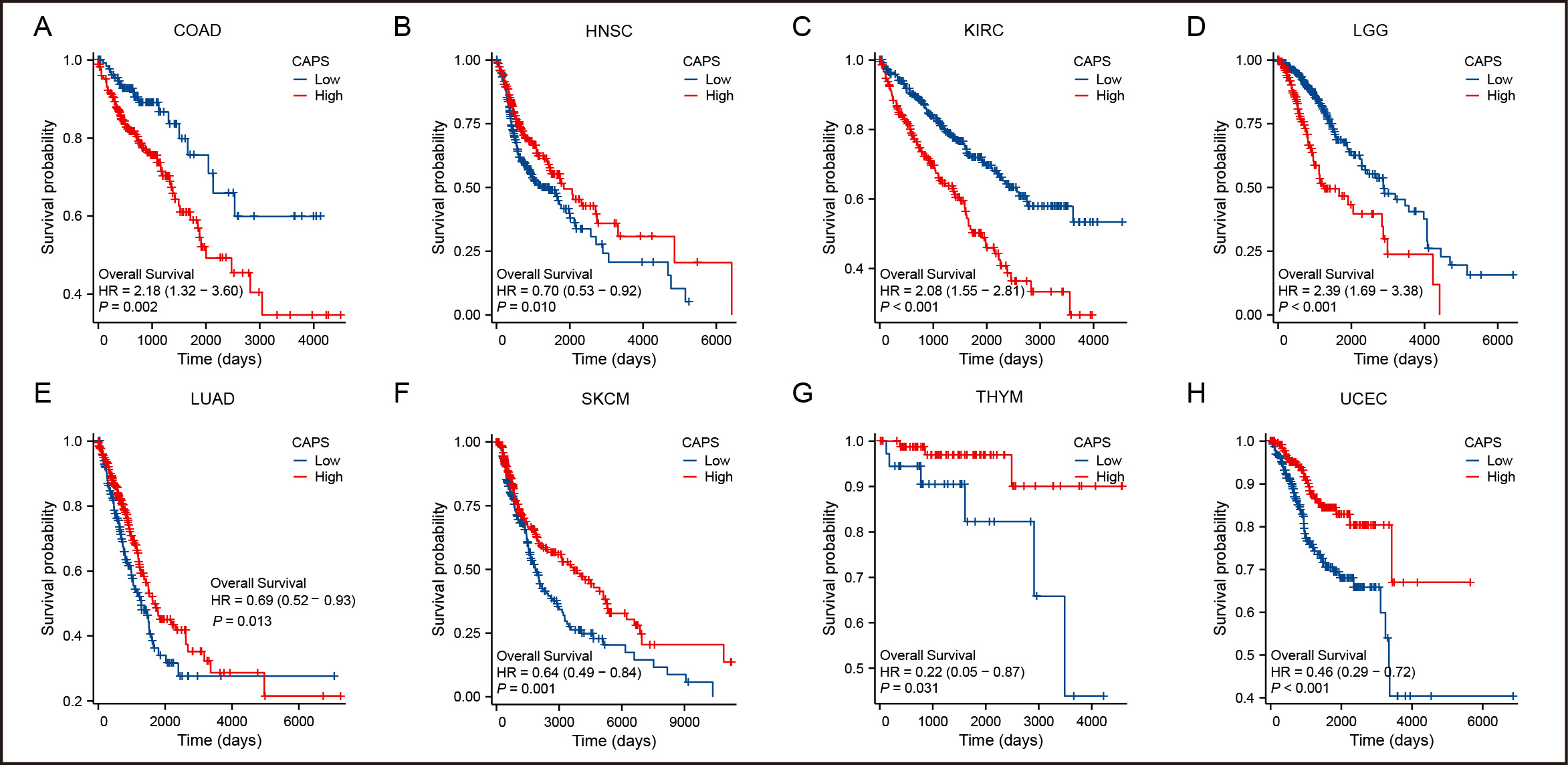

Supplement: Supplementary Figure 1 — Kaplan-Meier survival analyses of CAPS across TCGA cancer types. (A) Colon adenocarcinoma (COAD); (B) Head and neck squamous cell carcinoma (HNSC); (C) Kidney renal clear cell carcinoma (KIRC); (D) Lower-grade glioma (LGG); (E) Lung adenocarcinoma (LUAD); (F) Skin cutaneous melanoma (SKCM); (G) Thymoma (THYM); (H) Uterine corpus endometrial carcinoma (UCEC). [file Image1.jpeg]

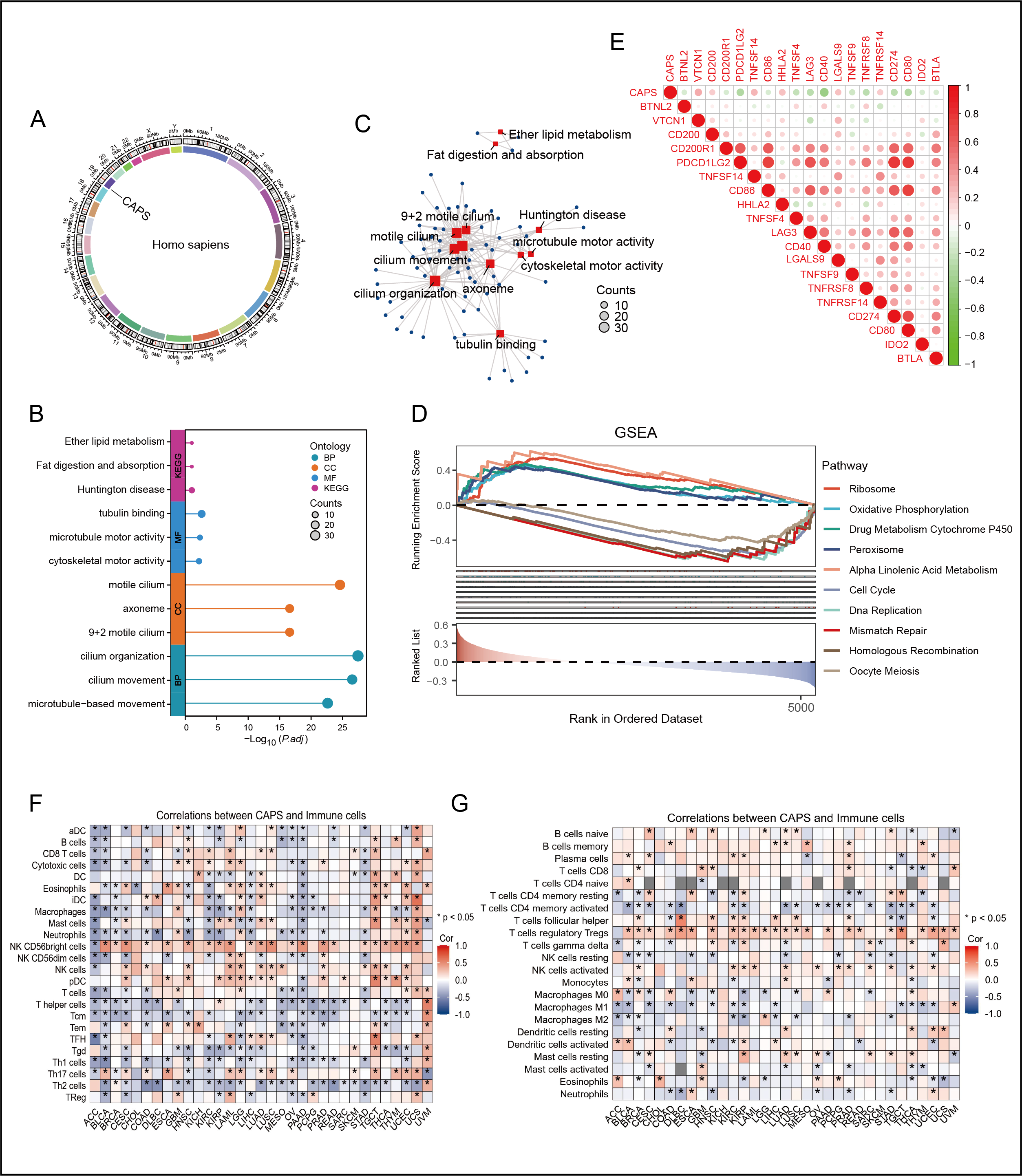

Supplement: Supplementary Figure 2 — Multidimensional analysis of CAPS functional features. (A) Precise chromosomal localization of CAPS at 19q13. (B-C) GO and KEGG enrichment analyses of CAPS-associated genes. (D) GSEA-based pathway enrichment of CAPS-associated genes. (E) Correlation between CAPS expression and canonical immune checkpoint molecules. (F) Pan-cancer infiltration of 24 immune cell types estimated by ssGSEA. (G) Pan-cancer infiltration of 22 immune cell types estimated by CIBERSORT. (* P< 0.05, ** P < 0.01, *** P< 0.001). (Abbreviations: GO, Gene Ontology; KEGG, Kyoto Encyclopedia of Genes and Genomes; GSEA, Gene Set Enrichment Analysis; ssGSEA, single-sample GSEA.). [file Image2.png]

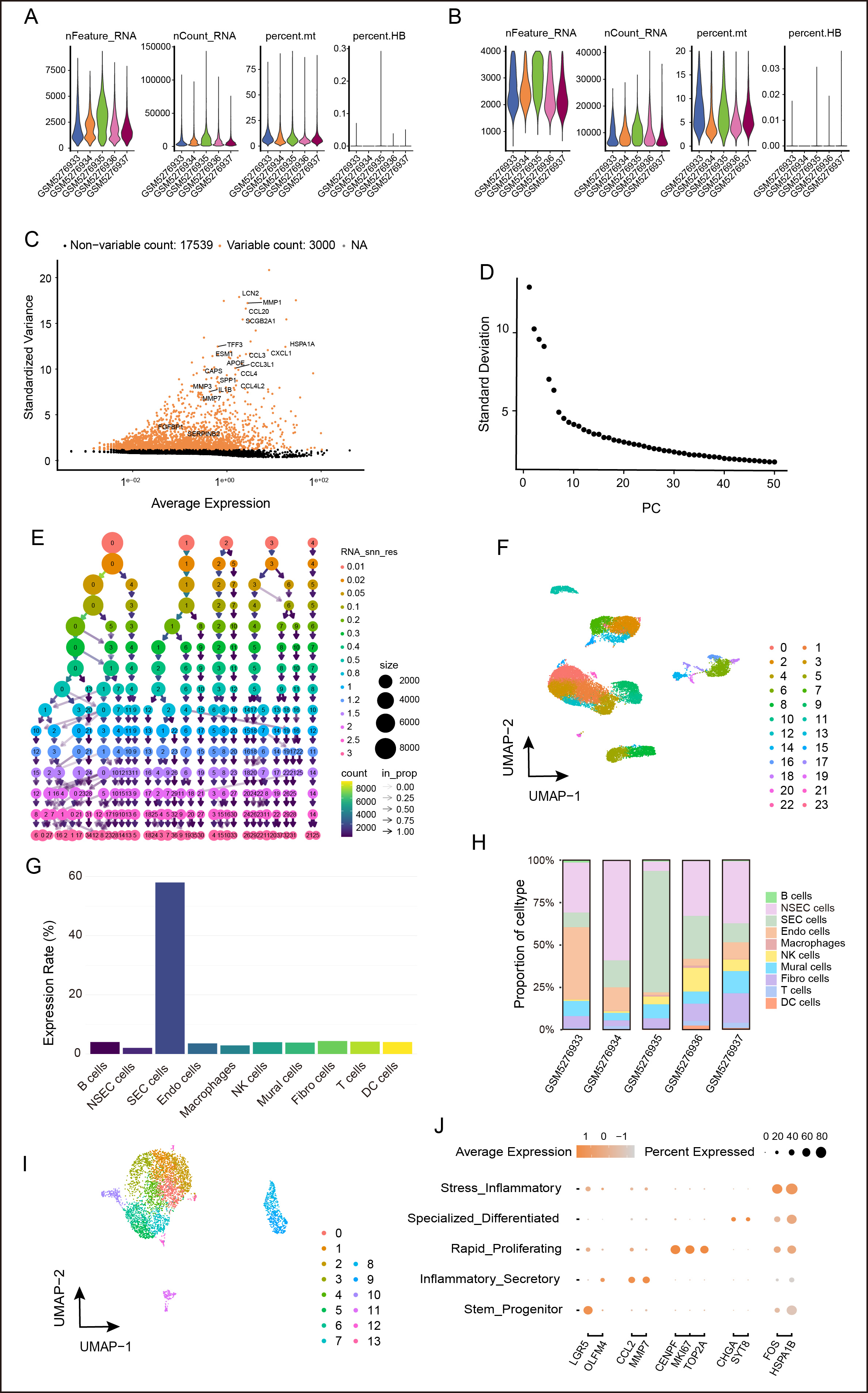

Supplement: Supplementary Figure 3 — Single-cell RNA-seq analysis workflow and validation of CAPS expression features. A-B. Quality control of raw single-cell RNA-seq samples (A, before QC; B, after QC). C. Selection of HVGs. D. Elbow plot assessing PC significance. E. Seurat clustering resolution tree. F. UMAP two-dimensional embedding based on the top 15 PCs, partitioning all cells into 24 clusters. G. Proportional distribution of cell types across different patient samples. H. Bar plot showing the proportion of CAPS-positive cells. I. Secondary clustering of SecEC; UMAP displays 14 subclusters. J. Subclustering and functional annotation of secretory endometrial epithelial cells (SeECs) (Abbreviations: QC, quality control; HVG(s), highly variable gene(s); PC(s), principal component(s); UMAP, Uniform Manifold Approximation and Projection; SecEC, secretory epithelial cells.). [file Image3.jpeg]

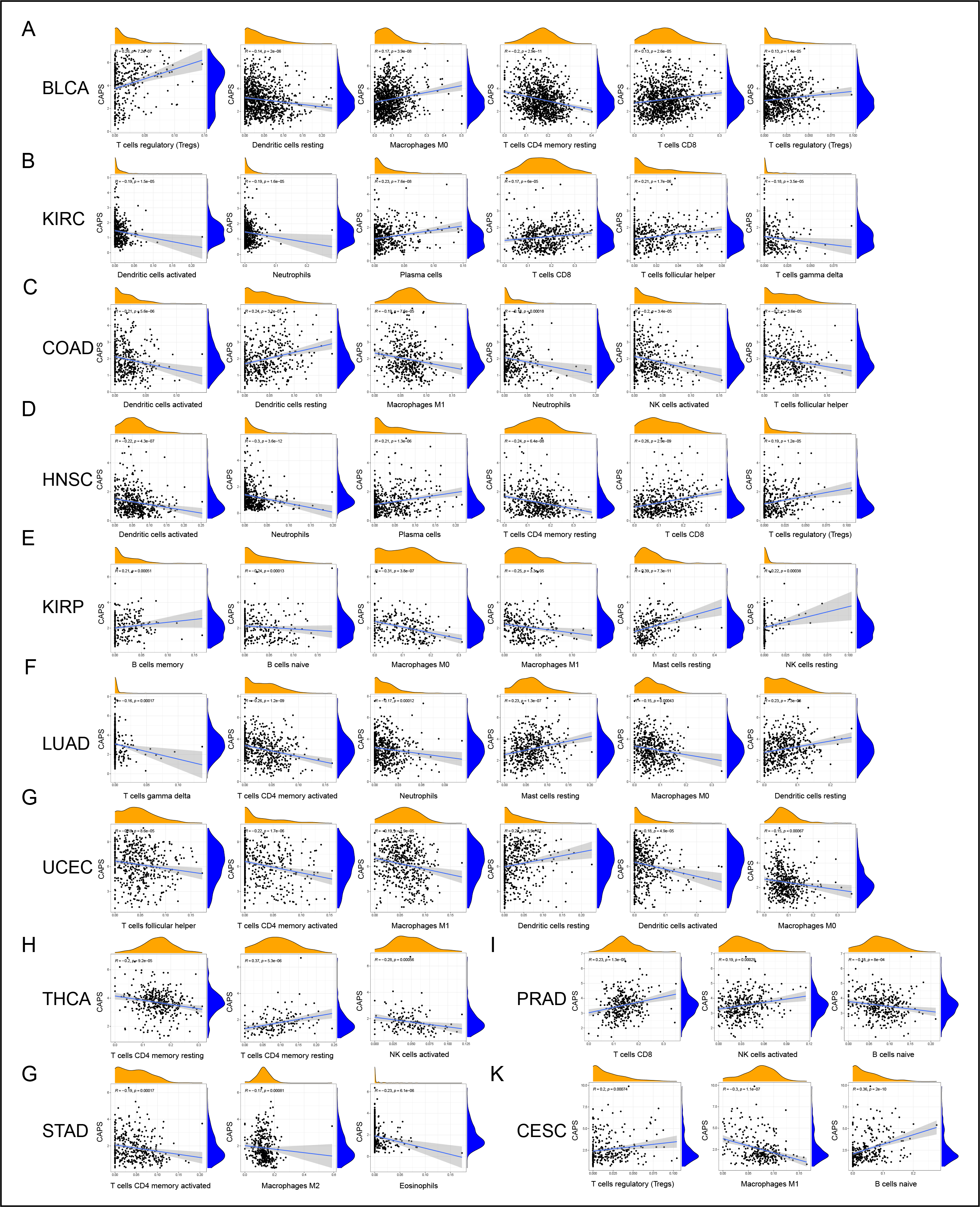

Supplement: Supplementary Figure 4 — Correlations between CAPS expression and immune/stromal cell infiltration across TCGA pan-cancer cohorts. (A-K) Cancer-type-specific analyses-including BLCA, KIRC, and COAD-show the association between CAPS expression and estimated levels of immune and stromal infiltration. [file Image4.png]
