# Supplementary material for: Pan-cancer multi-omics characterization of calcyphosine and its revealed links to the immune microenvironment and regulatory networks in endometrial carcinoma
Source: Front Immunol. 2025 Nov 26;16:1688606. doi: 10.3389/fimmu.2025.1688606 (PMC12689553; doi:10.3389/fimmu.2025.1688606)
Supplement: Supplementary Table 3 — Relative mRNA expression of CAPS in EC cell lines. [file Table2.docx]

| Patient | Cancer type | Tumor site | Histology | Stage | Age | BMI |
| --- | --- | --- | --- | --- | --- | --- |
| Patient 1 | Endometrial | Endometrium | Endometrioid | IA | 70 | 39.89 |
| Patient 2 | Endometrial | Endometrium | Endometrioid | IA | 70 | 30.5 |
| Patient 3 | Endometrial | Endometrium | Endometrioid | IA | 70 | 38.55 |
| Patient 4 | Endometrial | Endometrium | Endometrioid | IA | 49 | 55.29 |
| Patient 5 | Endometrial | Endometrium | Endometrioid | IA | 62 | 49.44 |

Supplementary Table 2. Clinical characteristics of EC samples from the GSE173682 single-cell RNA-seq dataset

Supplementary Table 3. Relative mRNA expression of CAPS in EC cell lines

| Relative mRNA expression of CAPS（25Q2） | EC Cell Line Name |
| --- | --- |
| 0.820 | EMTOKA |
| 0.867 | HEC50B |
| 1.260 | HEC151 |
| 1.327 | CCLFOVPA0001T |
| 1.421 | HTMMT |
| 1.468 | HEC265 |
| 1.518 | EFE184 |
| 1.519 | SNU685 |
| 1.650 | HEC116 |
| 1.664 | MFE319 |
| 1.683 | RL952 |
| 1.708 | HEC1A |
| 1.749 | HEC1B |
| 1.768 | HHUA |
| 1.786 | CX03 |
| 1.845 | SNU1077 |
| 1.846 | JHUEM7 |
| 2.095 | HEC6 |
| 2.200 | HEC1 |
| 2.344 | AN3CA |
| 2.363 | MFE296 |
| 2.381 | MFE280 |
| 2.414 | JHUEM2 |
| 2.713 | COLO684 |
| 2.760 | EN |
| 3.052 | HIRSBM |
| 3.077 | SNGM |
| 3.200 | HOUAI |
| 3.432 | HEC251 |
| 3.655 | HEC108 |
| 3.790 | KLE |
| 3.957 | JHUEM3 |
| 5.332 | ISHIKAWAHERAKLIO02ER |
| 5.592 | TEN |
| 8.117 | JHUEM1 |
| 8.916 | HEC59 |

Supplementary Table 4. Sequences of CAPS-targeting siRNAs and negative control

| siRNA label | Sequence (5′→3′) |
| --- | --- |
| Si1-CAPS | 5′-GGUUUUUCCGCCAACUAGACC-3′ |
| Si2-CAPS | 5′-GAAUUCCAGGACUACUACAGC-3′ |
| Si3-CAPS | 5′-GAGUUCGUGGCCAUGAUGACC-3′ |
| Si-NC | 5′-UUCUCCGAACGUGUCACGUTT-3′ |
